# An outbreak investigation of a large dengue fever outbreak in Somaliland, 2023–2024

**DOI:** 10.4102/jphia.v17i1.1842

**Published:** 2026-10-23

**Authors:** Hibo Asad, Amal Ali, Ahmed Hashi, Idhil Hirsi, Mahdi D. Bahdoon, Victor Del Rio Vilas, Oliver J. Brady, Hilary Bower, Farhana Haque, Ruoran Li, Mohamed A. Hergeye

**Affiliations:** 1 UK Public Health Rapid Support Team, UK Health Security Agency, London, United Kingdom; 2 Somaliland Ministry of Health Development, Hargeisa, Somaliland; 3 Somaliland National Public Health Laboratory, Hargeisa, Somaliland; 4 Department of Infectious Disease Epidemiology and Dynamics, Faculty of Epidemiology and Population Health, London School of Hygiene & Tropical Medicine, London, United Kingdom; 5 UK Public Health Rapid Support Team, Faculty of Epidemiology and Population Health, London School of Hygiene & Tropical Medicine, London, United Kingdom

**Keywords:** dengue, disease outbreaks, rapid diagnostic tests, epidemiology, Eastern Africa, humans

## Abstract

Somaliland experienced a large dengue fever outbreak between 2023 and 2024. National laboratory surveillance identified 4971 clinically suspected cases that underwent testing (‘tested probable cases’), of which 1703 were confirmed using rapid diagnostic tests. Three deaths were reported. Cases spanned five regions of Somaliland, with 74% in Marodijeh. As surveillance data were restricted to public-sector facilities, these findings represent a lower-bound estimate of true dengue transmission.

**Contribution:**

We highlight the utility of the new laboratory surveillance and the need for mitigating dengue risks amid climatic fluctuations.

## Background

Dengue fever (DF), transmitted primarily by the *Aedes aegypti* mosquito, has significantly impacted Eastern Africa and the Middle East since its re-emergence in the late 20th century, with three serotypes now circulating (Dengue virus, DENV-1, DENV-2 and DENV-3).^1,2,3^ Somaliland is a self-declared republic, a partially recognised state in the Horn of Africa that declared independence from Somalia in May 1991. Somaliland’s first reported DF outbreak occurred in its capital city, Hargeisa, during 1985–1987.^4^ No further outbreaks were reported^1^ until October 2022, when the Somaliland Ministry of Health Development (MoHD) declared a DF outbreak^5^ concurrent with outbreaks in neighbouring regions of Somalia.^6,7^ By February 2023, fever cases at public healthcare facilities in Somaliland declined and remained low (Appendix 1 Figure 1-A1), until 14 September 2023 when a new cluster of probable dengue patients was identified at a hospital near Hargeisa. Following confirmation by the National Public Health Laboratory (NPHL) using reverse transcriptase polymerase chain reaction (RT-PCR), MoHD declared a new DF outbreak on 03 October 2023 and initiated control measures, including spraying for vector reduction, risk communication and community engagement and training of healthcare personnel in clinical management. On 21 October 2023, MoHD requested assistance from the United Kingdom (UK) Public Health Rapid Support Team to conduct a joint epidemiologic investigation.

## Methods

### Case definition

A ‘probable DF case’ was defined as having fever and ≥ 2 of the following symptoms: nausea/vomiting, rash, aches and pains, tourniquet test positive, leukopenia, any warning sign,^8^ identified at a healthcare facility in Somaliland, with symptom onset between 01 September 2023 and 30 June 2024. A ‘confirmed case’ was a probable case with a positive result for immunoglobulin M (IgM) or non-structural protein 1 (NS1) antigen in a single serum sample using a rapid diagnostic test (RDT). Sample collection or reporting date was assumed to represent the symptom onset date if unavailable.

### Case finding and data source

We collected morbidity data from NPHL and mortality data from MoHD. From September 2023, NPHL procured a limited supply of dengue RDTs (SD Bioline Dengue Ag/Ab [NS1/IgM]) from the World Health Organization (WHO) and trained personnel in public-sector referral health centres and hospitals to test probable patients and report all tests to a newly established surveillance system with data on: date of sample collection, type and result of dengue test, patient’s gender, age, place of residence, presenting healthcare facility, presenting symptoms and date of symptom onset. Only probable cases that underwent laboratory testing were included in the system; thus, our study population consisted of only *tested probable cases (all ‘cases’), which were subsequently classified as confirmed (RDT positive) or non-confirmed (RDT negative)*. Data were received from seven of the 23 districts spanning five of Somaliland’s six regions (Figure 1a); Sool Region did not report dengue testing because of an unstable situation. The MoHD collated the number of dengue-related deaths retrospectively.

**FIGURE 1:**
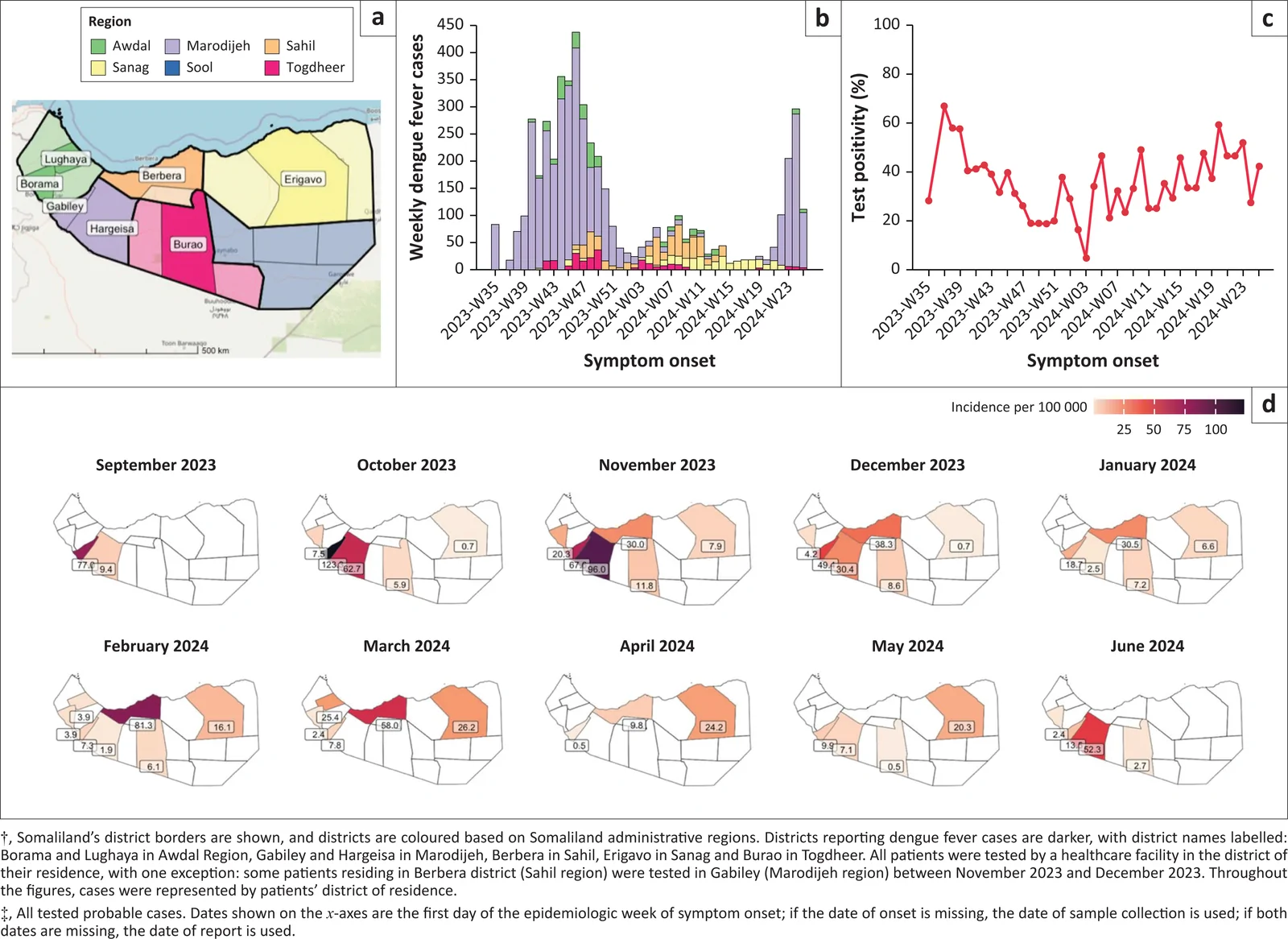
Distribution of tested dengue fever cases†, Somaliland, 01 September 2023 – 30 June 2024‡. (a) Somaliland’s district borders are shown, and districts are coloured according to Somaliland administrative regions. Districts reporting dengue fever cases are darker, with district names labelled: Borama and Lughaya in Awdal Region, Gabiley and Hargeisa in Marodijeh, Berbera in Sahil, Erigavo in Sanag and Burao in Togdheer. (b) Weekly dengue fever cases in a stacked bar chart for all Somaliland regions over the study period. (c) Weekly dengue fever test positivity trend over the study period. (d) Monthly incidence of dengue fever by districts in Somaliland.

### Data analysis

We report the attack rate (number of tested probable cases or confirmed cases/population based on the 2023 MoHD Population Estimation Survey, Appendix 1 Table 1-A1) and the case fatality rate (CFR) (number of dengue-related deaths/number of tested probable cases). All analyses were conducted using R version 4.5.0 (Vienna, Austria).

### Ethical considerations

Ethical approval was not required, as analyses were part of monitoring and the response to an ongoing outbreak. This investigation was conducted under the leadership and oversight of the Somaliland MoHD, which has the legal mandate for public health surveillance and outbreak response. Data were collected as part of routine public health monitoring, and all activities adhered to national guidelines on confidentiality, data protection and responsible use of health information. This article followed all ethical standards for research without direct contact with human or animal subjects.

## Results

Between 01 September 2023 and 30 June 2024, Somaliland NPHL documented 4971 tested probable DF cases, with 1703 (34.2%) confirmed by RDT (Table 1), and a small subset (*n* = 12) underwent RT-PCR at the start of the outbreak (2 negatives and 10 positives). The outbreak predominantly affected younger adults and children (median age of all cases: 23 years; interquartile range: 10–35). Overall DF attack rate was 109 cases per 100 000 population over the 10-month period, with 37.2 confirmed cases per 100 000. Ministry of Health Development (MoHD) reported three DF-related deaths (CFR 0.06%).

**TABLE 1:** Dengue fever patient characteristics by rapid diagnostic test† positivity, Somaliland, 01 September 2023 – 30 June 2024.

Patient characteristics	Tested probable cases (all cases in NPHL)				Confirmed cases (RDT† positive)				Test positivity	
	*n*	col %	Median	IQR	*n*	col %	Median	IQR	%	*p (χ*^2^ *test)*
**Overall**	4971	100	-	-	1703	100	-	-	34.3	-
**Attack rate (per 100 000)September 2023 – June 2024**	108.6	-	-	-	37.2	-	-	-	-	-
**Age (years)**	-	-	23	10–35	-	-	24	12–35	-	-
**Age group (years)**	-	-	-	-	-	-	-	-	-	*0.07*
0–4	744	15.0	-	-	232	13.6	-	-	31.2	-
5–9	463	9.3	-	-	140	8.2	-	-	30.2	-
10–19	790	15.9	-	-	279	16.4	-	-	35.3	-
20–29	1201	24.2	-	-	413	24.3	-	-	34.4	-
30–39	716	14.4	-	-	241	14.2	-	-	33.7	-
40–49	391	7.9	-	-	147	8.6	-	-	37.6	-
50–59	251	5.0	-	-	101	5.9	-	-	40.2	-
60+	318	6.4	-	-	112	6.6	-	-	35.2	-
*Missing*	*97*	*2.0*	-	-	*38*	*2.2*	-	-	*39.2*	-
**Gender**	-	-	-	-	-	-	-	-	-	*0.3*
Female	2412	48.5	-	-	846	49.7	-	-	35.1	-
Male	2557	51.4	-	-	857	50.3	-	-	33.5	-
*Missing*	*2*	*< 0.1*	-	-	*0*	*0*	-	-	*0*	-
**Region (districts) of residence** ‡	-	-	-	-	-	-	-	-	-	*< 0.001*
Awdal (Borama, Lughaya)	266	5.4	-	-	17	1.0	-	-	6.4	
Marodijeh (Gabiley, Hargeisa)	3674	73.9	-	-	1368	80.3	-	-	37.2	-
Sahil (Berbera)	479	9.6	-	-	168	9.9	-	-	35.1	-
Sanag (Erigavo)	313	6.3	-	-	103	6.0	-	-	32.9	-
Togdheer (Burao)	239	4.8	-	-	47	2.8	-	-	19.7	-
**Period§**	-	-	-	-	-	-	-	-	-	*< 0.001*
September 2023 – October 2023	1278	25.7	-	-	527	30.9	-	-	40.9	-
November 2023 – December 2023	2071	41.7	-	-	596	35.0	-	-	29.0	-
January 2024 – February 2024	484	9.7	-	-	135	7.9	-	-	28.6	-
March 2024 – April 2024	329	6.6	-	-	117	6.9	-	-	35.1	-
May 2024 – June 2024	809	16.3	-	-	328	19.3	-	-	40.2	-

NPHL, National Public Health Laboratory; IQR, interquartile range; RT-PCR, reverse transcriptase polymerase chain reaction; IgM, immunoglobulin M; RDT, rapid diagnostic test; col %, column %.

† , All samples were tested with RDTs, which detect the NS1 antigen, the IgM antibodies or a combination of NS1 and IgM. RDT results of 12 samples (2 negative and 10 positive) at the beginning of the outbreak were confirmed using RT-PCR;

‡ , All patients were tested by a healthcare facility in the district of their residence, with one exception: some patients residing in Berbera district (Sahil region) were tested in Gabiley (Marodijeh region) between November 2023 and December 2023;

§ , Case period is calculated based on the date of symptom onset; if the date of symptom onset is missing, the date of sample collection is used; if the date of sample collection is missing, the date of report is used.

The outbreak peaked in mid-November 2023 with 436 new cases per week, predominantly from the capital region of Marodijeh (Figure 1a–b). Cases dropped to fewer than 100 weekly cases in early 2024. Between January 2024 and May 2024, the northeastern regions of Sahil and Sanag were most affected. Marodijeh experienced a second surge in June 2024. Throughout the outbreak, Togdheer and Awdal regions reported only intermittent cases.

Patient characteristics varied by time and region (Appendix 1 Table 2-A1). Marodijeh saw older cases in the 2024 wave than in the 2023 wave (median age 25 years vs 18 years, Mann–Whitney test *p* < 0.001). Other regions reported median ages ranging from 25 years to 32 years. Gender distributions were relatively balanced (48.5% female). Similar patterns were observed among confirmed cases (Appendix 1 Table 3-A1).

Rapid diagnostic testing began in September 2023 in Marodijeh and expanded to all regions except Sool by November 2023. During October 2023 – November 2023, 84% of febrile patients reported through public facilities were tested (Appendix 1 Figure 1-A1). Overall, one third of probable cases tested positive for dengue (Table 1, Figure 1c). Older patients were more likely to test positive (chi-squared trend test: *p* = 0.004). Test positivity exceeded 40% during the first 2 months of the outbreak and as cases resurged in May 2024 – June 2024. Frequent testing gaps were observed in Awdal, Sahil, Sanag and Togdheer regions (Appendix 1 Figure 2-A1), coinciding with reported shortages of RDT kits.

## Discussion

This investigation describes extensive and recurring dengue transmission in Somaliland from September 2023 to June 2024 with 4971 tested probable cases and three reported deaths. Concurrent transmission was reported in neighbouring Ethiopia^9,10^ and Yemen.^2^ While clinical features of the second wave of this outbreak have previously been described,^11^ here we show that transmission was sustained across multiple waves for almost a year and spread across multiple regions. The presence of cases across all age groups, matching the population’s age structure, and the low CFR (0.06%) suggest that most cases represent primary dengue infections; however, this remains a hypothesis in the absence of serological evidence. As subsequent infections with different dengue serotypes are more likely to result in serious clinical manifestations, this leaves Somaliland vulnerable to more severe future outbreaks.

Recent extreme climatic conditions in Eastern Africa may have contributed to changes in dengue risk, as high rainfall or droughts that lead to standing water in open containers increase *Aedes Aegypti* egg-laying habitat.^12^ Historically, Somaliland observes two rainy seasons: March–June [*gu*] and October–November [*deyr*]. During the 2023 *deyr*, rains were exceptionally heavy,^13^ coinciding with the outbreak’s onset. Rainfall deficits in the 2024 *gu* season^14^ coincided with a new peak in dengue cases in June. Although causality cannot be inferred solely from the observed temporal overlap between rainfall patterns and dengue incidence, these fluctuations underscore the urgent need for integrated surveillance that combines entomological and meteorological data to better understand dengue transmission dynamics in the region. Drought and flood response planning in Somaliland must specifically address potential increases in dengue risk and identify strategies for mitigation.

The investigation has several limitations. Reliance on NPHL’s surveillance of laboratory-tested probable cases led to underreporting of milder cases, cases from areas with limited healthcare access (one-third of the population lives in nomadic settlements, Appendix 1 Table 4-A1) and cases presenting to primary-care facilities, which had very limited or no access to case detection training or RDT kits. The changing availability of RDT affected clinical criteria for testing; no data were available to estimate the testing fraction over time. Syndromic surveillance will complement RDT testing and mitigate this bias in resource-constrained settings. Only 12 cases were tested by RT-PCR; reliance on RDT testing may have introduced diagnostic misclassification, as IgM cross-reactivity with other flaviviruses and antibody persistence can result in false-positive RDTs, leading to an overestimation of the true burden of dengue among tested cases. The lack of viral serotyping precludes further insights into transmission dynamics and disease severity. Additionally, NPHL did not have access to private facility data; the 2020 Somaliland Health and Demographic Survey reported that residents were 50% more likely to visit private facilities than public ones when sick, and that healthcare-seeking patterns varied by urbanicity, region and wealth.^15^ Our figures likely represent a lower-bound estimate of the scale of the DF outbreak in Somaliland. Demographic differences between patients in public and private facilities further constrained the representativeness of NPHL data. These limitations preclude interpreting regional heterogeneity in disease attack rates and test positivity rates as definitive indicators of dengue transmission intensity. Caution is also required when assessing transmission dynamics given the incompleteness of symptom onset data; nonetheless, sensitivity analyses limited to cases with complete symptom onset dates revealed no substantial distortions to reported trends (Appendix 1 Figure 3-A1). Furthermore, the retrospective reporting of dengue-related deaths and the lack of information on patient outcome in laboratory surveillance may have resulted in an underestimation of the true CFR in public facilities; a CFR of 0.06% is low compared with those reported by some other regions (0.01% – 1%).^1^ Lastly, the absence of vector and serotype surveillance and reporting of tests for other pathogens of probable cases who were RDT negative prevented more comprehensive analyses of outbreak dynamics and potential changes in severity.

Despite these limitations, the investigation demonstrates the utility of the new laboratory surveillance system in Somaliland during its first year of operation. Strengthening an integrated DF surveillance system will not only preserve institutional memory but also provide insights into how socioeconomic and climatic factors amplify dengue risks. Given the large nomadic population, understanding the spatiotemporal patterns of disease burden is crucial for informing more tailored interventions to mitigate dengue transmission.

## Figures and Tables

**FIGURE 1-A1:**
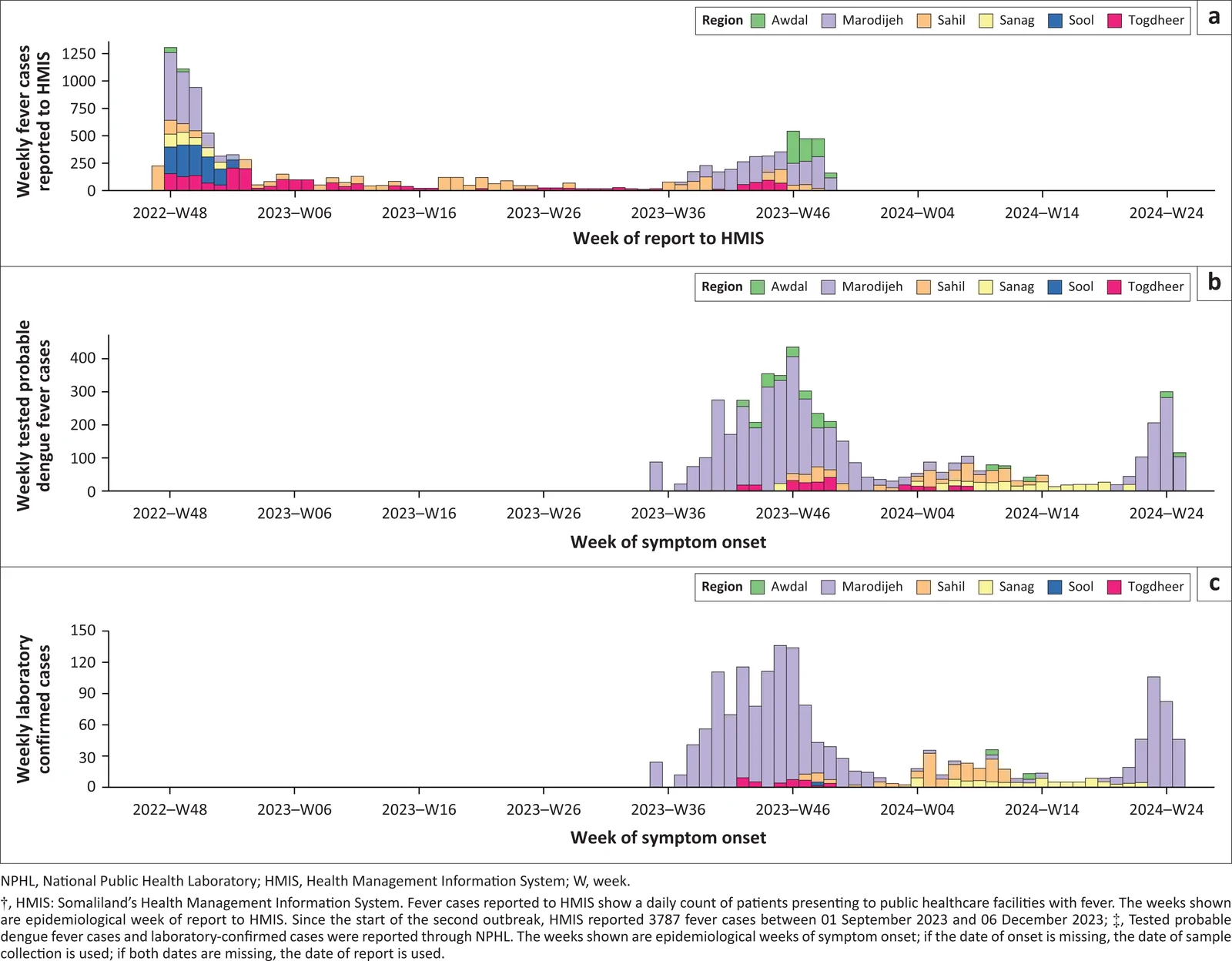
Weekly number of fever cases reported to Somaliland’s Health Management Information System†, tested probable dengue fever cases and laboratory-confirmed cases‡, Somaliland, 26 November 2022 – 30 June 2024. (a) Fever cases reported to Somaliland’s Health Management Information System. (b) Tested probable dengue fever cases. (c) Laboratory-confirmed cases.

**FIGURE 2-A1:**
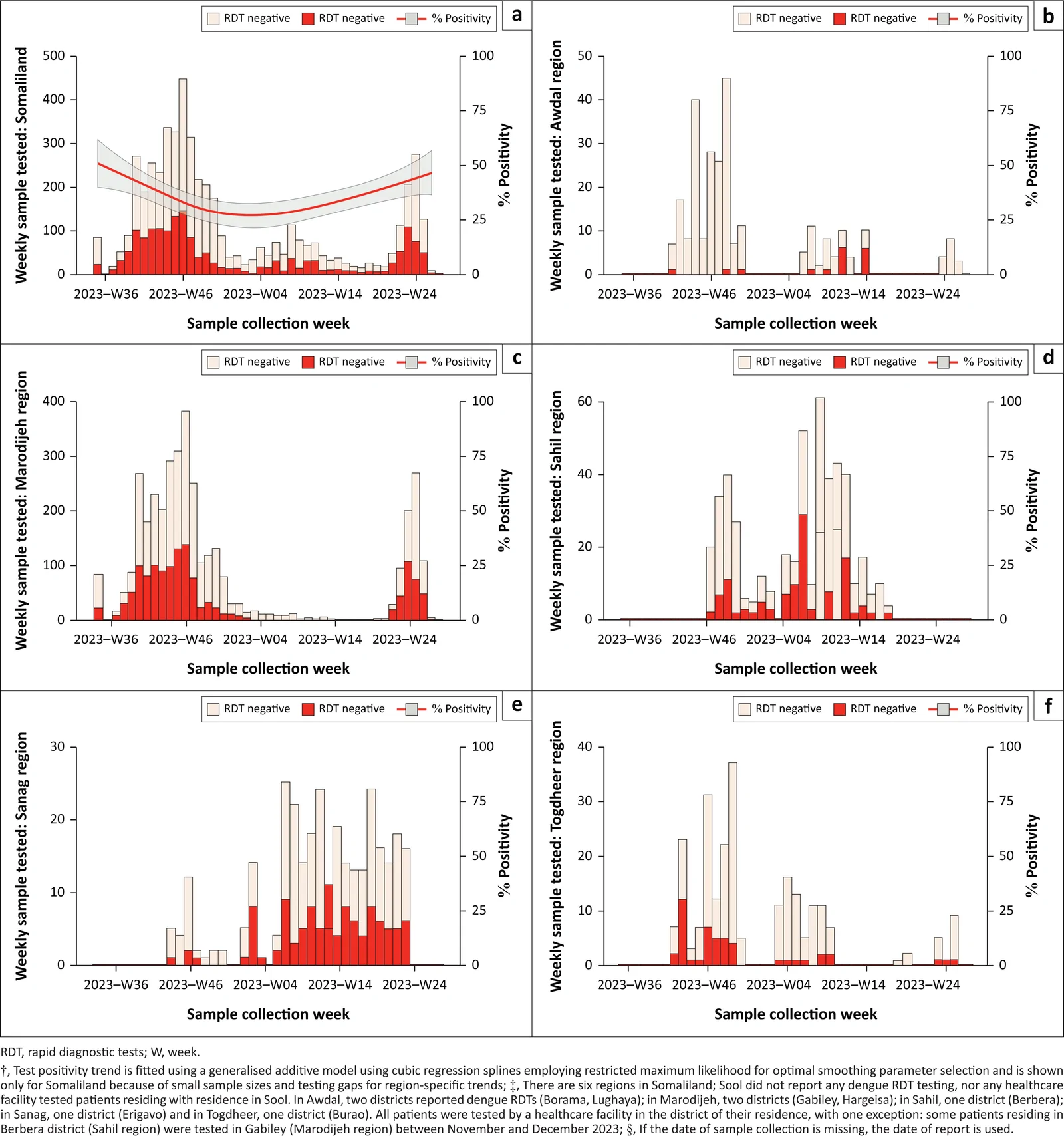
Trends in volume and positivity† of laboratory samples tested for dengue using rapid diagnostic tests, by region of patient residence‡, Somaliland, 01 September 2023 – 30 June 2024§. (a) Somaliland; (b) Region: Awdal; (c) Region: Marodijeh; (d) Region: Sahil; (e) Region: Sanag and (f) Region: Togdheer.

**FIGURE 3-A1:**
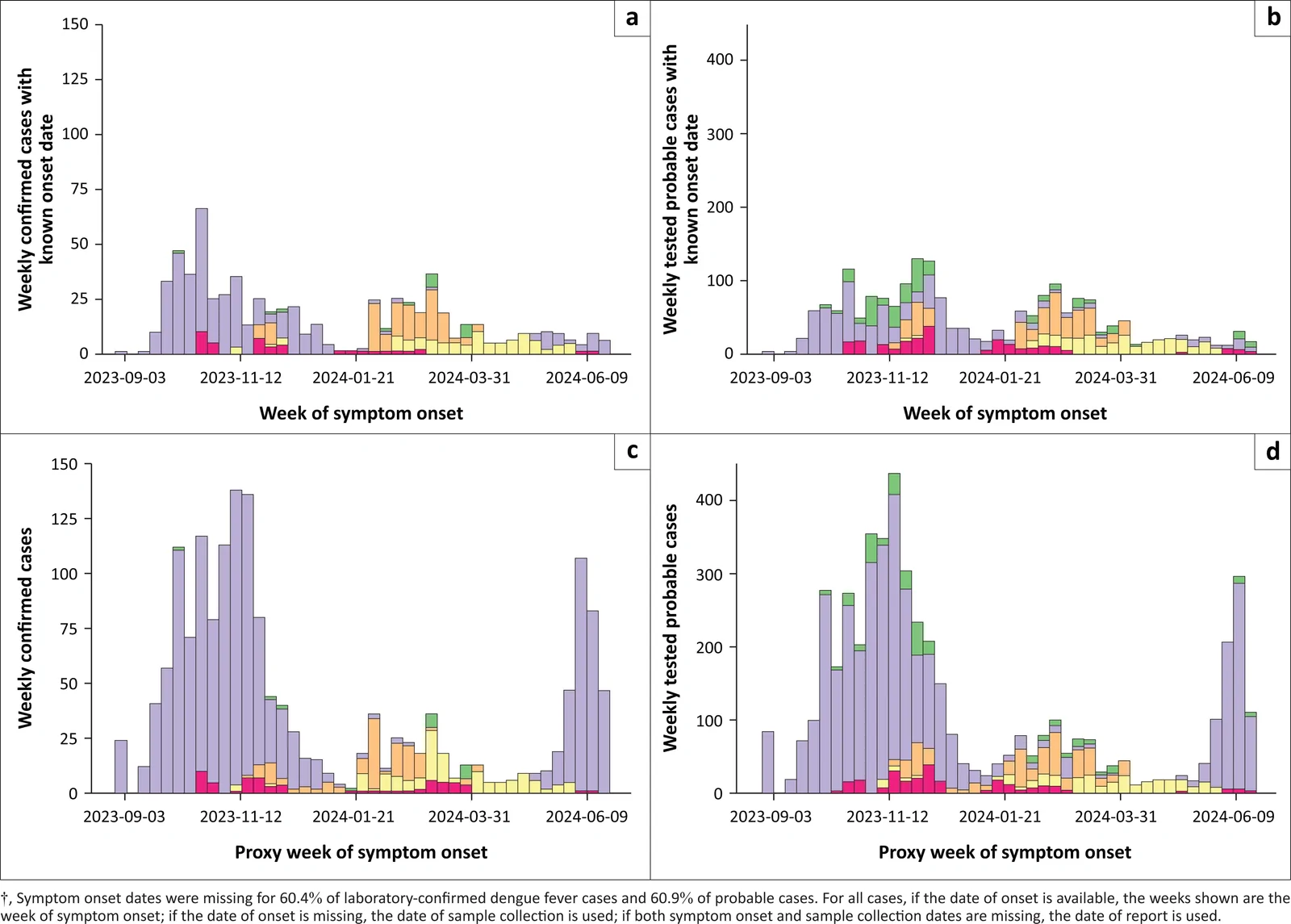
Weekly number of dengue fever cases by case status and certainty of symptom onset date†, Somaliland, 01 September 2023 – 30 June 2024. (a) Confirmed cases, known onset week (*n* = 675), (b) Confirmed cases, all cases (*n* = 1703), (c) Tested probable cases, known onset week (*n* = 1944), (d) Tested probable cases, all cases (*n* = 4971).

## Appendix 1

**TABLE 1-A1:** Population size estimates in Somaliland’s districts, 2023.

Region	District	Population size estimates, 2023
Awdal	Baki	76 759
	Borama	614 919
	Lughaya	51 172
	Zeila	136 459
Marodijeh	Baligubadle	54 905
	Gabiley	192 168
	Hargeisa	1 125 553
Sahil	Berbera	193 198
	Sheikh	95 157
Sanag	Badhan	255 585
	El Afweyn	149 091
	Erigavo	305 282
Sool	Hudun	64 083
	Las Anod	281 965
	Taleh	81 172
Togdheer	Aynabo	117 100
	Buhodle	81 069
	Burao	558 476
	Odwayne	144 123

*Source:* Population estimates based on Somaliland Ministry of Health Development Population Estimation Survey, 2023

**TABLE 2-A1:** Characteristics of tested dengue fever cases* by region of residence, Somaliland, 01 September 2023 – 30 June 2024.

Patient characteristics	All cases†		Region of residence											
			Marodijeh, 2023‡		Marodijeh, 2024‡		Awdal		Sahil		Sanag		Togdheer	
	*n*	%	*n*	col %	*n*	col %	*n*	col %	*n*	col %	*n*	col %	*n*	col %
All cases	**4971**	-	2845	-	829	-	266	-	479	-	313	-	239	-
Attack rate (per 100 000)01 September 2023 – 30 June 2024	**108.6**	-	207.3	-	60.4	-	30.3	-	166.1	-	44.1	-	26.5	-
**Period¶**														
September 2023 – October 2023	**1278**	**25.7**	1197	42.1	-	-	46	17.3	0	0	≤ 5§	-	33	13.8
November 2023 – December 2023	**2071**	**41.7**	1648	57.9	-	-	151	56.8	132	27.6	26	8.3	114	47.7
January 2024 – February 2024	**484**	**9.7**	-	-	99	11.9	26	9.8	216	45.1	69	22.0	74	31.0
March 2024 – April 2024	**329**	**6.6**	-	-	16	1.9	28	10.5	131	27.3	154	49.2	0	0
May 2024 – June 2024	**809**	**16.3**	-	-	714	86.1	15	5.6	0	0	62	19.8	18	7.5
**Age group (years)**														
0–4	**744**	**15.0**	630	22.1	94	11.3	9	3.4	6	1.3	≤ 5	-	≤ 5	-
5–9	**463**	**9.3**	350	12.3	73	8.8	17	6.4	≤ 5	-	≤ 5	-	17	7.1
10–19	**790**	**15.9**	479	16.8	129	15.6	52	19.5	92	19.2	13	4.2	25	10.5
20–29	**1201**	**24.2**	535	18.8	203	24.5	78	29.3	202	42.2	106	33.9	77	32.2
30–39	**716**	**14.4**	312	11.0	121	14.6	54	20.3	95	19.8	88	28.1	46	19.2
40–49	**391**	**7.9**	195	6.9	70	84	21	7.9	28	5.8	55	17.6	22	9.2
50–59	**251**	**5.0**	144	5.1	53	6.4	14	5.3	10	2.1	27	8.6	≤ 5	-
60+	**318**	**6.4**	180	6.3	85	10.3	21	7.9	≤ 5	-	20	6.4	8	3.3
Missing	**97**	**2.0**	20	0.7	≤ 5	-	0	0	38	7.9	0	0	38	15.9
**Gender**														
Female	**2412**	**48.5**	1375	48.3	439	53.0	131	49.2	200	41.8	150	47.9	117	49.0
**Delay from symptom onset to sample collection†† (days)**	-	-	-	-	-	-	-	-	-	-	-	-	-	-
Missing symptom onset date	**3027**	**60.9**	2206	77.5	683	82.4	0	0	80	16.7	20	6.4	38	15.9

Note: All cases: Age (years) – Median = 23; IQR = 10–35. Delay from symptom onset to sample collection (days) – Mean = 3.61; s.d. = 4.35. Region of residence - Age (years): Marodijeh, 2023 – Median = 18, IQR = 5–32; Marodijeh, 2024 – Median = 25, IQR = 13–40; Awdal – Median = 26, IQR = 18–35; Sahil – Median = 25, IQR = 20–30; Sanag – Median = 32, IQR = 24–42; Togdheer – Median = 25, IQR = 21–34. Delay from symptom onset to sample collection (days): Marodijeh, 2023 – Mean = 3.49, s.d. = 3.27; Marodijeh, 2024 – Mean = 3.93, s.d. = 3.59; Awdal – Mean = 1.12, s.d. = 1.78; Sahil – Mean = 3.53, s.d. = 2.84; Sanag – Mean = 6.88, s.d. = 7.88; Togdheer – Mean = 2.46, s.d. = 1.98.

s.d., standard deviation; IQR, interquartile range.

† , All tested probable dengue fever cases;

‡ , The two calendar years correspond to the two dengue waves in Marodijeh;

§ , Counts ≤ 5 are suppressed to preserve patient confidentiality;

¶ , Case period is calculated based on the date of symptom onset; if the date of symptom onset is missing, the date of sample collection is used; if the date of sample collection is missing, the date of report is used;

†† , If the date of sample collection is missing, the date of report is used.

**TABLE 3-A1:** Characteristics of confirmed dengue fever cases†, Somaliland regions, 01 September 2023 – 30 June 2024.

Patient characteristics	All confirmed cases		Region of residence											
			Marodijeh, 2023‡		Marodijeh, 2024‡		Awdal		Sahil		Sanag		Togdheer	
	*n*	%	*n*	%	*n*	%	*n*	%	*n*	%	*n*	%	*n*	%
Confirmed cases	**1703**	-	1052	-	316	-	17	-	168	-	103	-	47	-
Attack rate (per 100 000) 01 September 2023 – 30 June 2024	**37.2**	-	76.6	-	23.0	-	1.9	-	58.3	-	14.5	-	5.2	-
**Period¶**														
September–October 2023	**527**	**30.9**	511	48.2	-	-	≤ 5§	-	0	0	0	0	15	31.9
November–December 2023	**596**	**35.0**	541	51.4	-	-	≤ 5	-	27	16.1	≤ 5	-	22	46.8
January–February 2024	**135**	**7.9**	-	-	12	3.8	≤ 5	-	89	53.0	24	23.3	8	17.0
March–April 2024	**117**	**6.9**	-	-	≤ 5		12	70.6	52	31.0	52	50.5	0	0
May–June 2024	**328**	**19.3**	-	-	303	95.9	0	0	0	0	23	22.3	≤ 5	-
**Age (years)**	-	-	-	-	-	-	-	-	-	-	-	-	-	-
Age group: 0–4	**232**	**13.6**	195	18.5	34	10.8	≤ 5	-	≤ 5	-	0	0	0	0
5–9	**140**	**8.2**	108	10.3	25	7.9	0	0	≤ 5	-	≤ 5	-	≤ 5	-
10–19	**279**	**16.4**	182	17.3	45	14.2	≤ 5	-	41	24.4	≤ 5	-	≤ 5	-
20–29	**413**	**24.3**	205	19.5	87	27.5	≤ 5	-	65	38.7	36	35.0	15	31.9
30–39	**241**	**14.2**	128	12.2	49	15.5	≤ 5	-	28	16.7	27	26.2	≤ 5	
40–49	**147**	**8.6**	90	8.6	23	7.3	≤ 5	-	≤ 5	-	18	17.5	7	14.9
50–59	**101**	**6.6**	65	6.2	21	6.6	≤ 5	-	≤ 5	-	9	8.7	≤ 5	-
60+	**112**	**2.2**	70	6.7	32	10.1	0	0	0	0	7	6.8	≤ 5	-
Missing	**38**	**2.2**	9	0.9	0	0	0	0	21	12.5	0	0	8	17.0
**Gender**														
Female	**846**	**49.7**	526	50.0	166	52.5	8	47.1	71	42.3	54	52.4	21	44.7
**Delay from symptom onset to sample collection†† (days)**	-	-	-	-	-	-	-	-	-	-	-	-	-	-
Missing	**1028**	**60.4**	707	67.2	271	85.8	0	0	32	19.0	10	9.7	8	17.0

Note: All cases: Age (years) – Median = 24; IQR = 12–35. Delay from symptom onset to sample collection (days) – Mean = 3.62; s.d. = 3.04. Region of residence – Age (years): Marodijeh, 2023 – Median = 20, IQR = 7–35; Marodijeh, 2024 – Median = 25.5, IQR = 15–37; Awdal – Median = 30, IQR = 25–40; Sahil – Median = 24, IQR = 19–30; Sanag – Median = 32, IQR = 24–42; Togdheer – Median = 25, IQR = 20–42. Delay from symptom onset to sample collection (days): Marodijeh, 2023 – Mean = 3.59, s.d. = 3.55; Marodijeh, 2024 – Mean = 4.33, s.d. = 2.24; Awdal – Mean = 3.47, s.d. = 1.42; Sahil – Mean = 3.42, s.d. = 1.58; Sanag – Mean = 4.18, s.d. = 3.38; Togdheer – Mean = 2.44, s.d. = 1.85.

s.d., standard deviation; IQR, interquartile range; IgM, immunoglobulin M; RDT, rapid diagnostic tests; RT-PCR, reverse transcriptase polymerase chain reaction.

† , All cases were tested with rapid diagnostic tests, which detect the NS1 antigen, the IgM antibodies or a combination of NS1 and IgM. RDT results of 10 positive samples at the beginning of the outbreak were confirmed by RT-PCR;

‡ , The two calendar years correspond to the two dengue waves in Marodijeh;

§ , Counts ≤ 5 are suppressed to preserve patient confidentiality;

¶ , Case period is calculated based on the date of symptom onset; if the date of symptom onset is missing, the date of sample collection is used; if the date of sample collection is missing, the date of report is used;

†† , If the date of sample collection is missing, the date of report is used.

**TABLE 4-A1:** Somaliland’s population by type of residence, 2016.

Variable	Region		Population by type of residence (row %)			
	Somaliland	Somalia	Nomadic	IDP camps	Urban	Rural
**Region-specific estimates**	Awdal	Awdal	34.7	1.2	42.8	21.4
	Marodijeh + Sahil	W. Galbeed	20.6	3.6	64.6	11.2
	Togdheer	Togdheer	21.4	3.6	67.1	8.0
	Sool	Sool	57.3	1.5	37.0	4.3
	Sanag	Sanaag	64.8	0.2	29.4	5.7

**Total Somaliland†**	-	-	**33.7**	**2.4**	**52.9**	**11.0**

*Source:* Population estimates from the UNFPA (2016) Population Composition and Demographic Characteristics of the Somali People, Volume 2, Figure 19, page 22

IDP, internally displaced population.

† , Calculated based on the region-specific proportion of population by type of residence (*Figure 19, p. 22*) and the population size as a proportion of the total Somali population (*Table 2, p. 20*).
